# Analysis of the psychometric properties of a banking instrument applied to the health sector

**DOI:** 10.4317/jced.59840

**Published:** 2022-12-01

**Authors:** María-José Muñoz-Leal, Josep-María Ustrell-Torrent, Marco Ferrari

**Affiliations:** 1 Licenciada en Odontología. Magister en Administración de Negocios. Leal University of Siena; 2 MD, PhD, Full Prof. Departament of Odontostomatology. University of Barcelona. Oral Health and Masticatory System Group (IDIBELL), Spain; 3 MD, DMD, Ph D. Profesor Titular y Director del Departamento de Prostodoncia y Materiales Dentales. Decano de la Facultad de Medicina Dental. University of Siena

## Abstract

**Background:**

Different studies have shown the importance of corporate reputation, corporate image and corporate identity and how they are present in the healthcare field. These studies show that these terms establish reputation as a socially constructed asset, which has been gaining relevance in organizations due to its increasing importance as a strategic resource. The aim of this study is to analyze the psychometric properties of an instrument used to measure reputation in the banking field, in its Spanish version, adapted to the health sector, thus determining whether it is a reliable and valid instrument to be used in future research on samples of users in the health sector.

**Material and Methods:**

In a pilot phase, the survey was applied to 97 patients. Then, the restructured instrument was applied to a sample of 323 men and women who attended the Dental Clinic of the Andrés Bello University, Concepción (Chile). During the pilot phase and application of the instrument, two exploratory factor analyses, Bartlett’s test, the Kaiser-Meyer-Olkin coefficient (KMO), the Principal Component Analysis (PCA) and the Varimax method with Kaiser normalization were applied. During the applicability analysis phase of the instrument, two confirmatory factor analyses were also applied for Relational Commitment (RC), Behavioral Intention (BI) and Relational Value (RV).

**Results:**

According to the results obtained, the instrument initially created by Khan, Kadir and Wahab for the banking area can be considered reliable and solid for estimating reputation in the health area. The adaptation made to the instrument is considered to exhibit appropriate psychometric properties consistent with the original instrument.

**Conclusions:**

Measuring relational value can go a long way in determining the future behavior of users who are accustomed to visiting an organization associated with the health sector.

** Key words:**Reputation, healthcare services, healthcare marketing, reputational value, relational value.

## Introduction

Corporate Reputation has acquired greater prominence within organizations, which is evident in the growing number of investigations focused on it, both from the business and academic point of view (1-3) and which necessarily go hand in hand with the development of methodologies that allow its measurement (4).

Organizations are aware of the importance of intangible values, and how these can have an impact on the economic valuation that companies can have thanks to corporate reputation (5), thus forming part of the culture of continuous improvement of many of them. That is why many organizations resort to instruments that allow them to obtain valuable information to incorporate it in their internal processes, in addition to being able to compete with other companies that use the same measurement standards, being comparable among them. In this way, rankings arise where consumers can have information about organizations, many of them increasingly aware of aspects such as social responsibility, the link with the environment, the work environment, among others that are generally measured by existing instruments today (6,7).

Every organization has two types of assets: tangible and intangible. The former are related to those elements such as buildings, equipment, materials for the production of products, among others. Intangible assets correspond to the brand, corporate reputation, patents, etc., which add value to the organization. It is increasingly common to find companies that are giving greater relevance to their intangible assets, since it has been proven how these can have an impact on the improvement of productivity and financial statements. Hence the importance of being able to measure and evaluate the impact of intangibles in organizations (8).

In a world of increasing globalization and competition, companies are focusing their efforts on differentiating themselves through aspects that are more sustainable over time, which is why intangible assets play a fundamental role in this regard. According to Porter’s competitive advantage model, there are two basic types of competitive advantage: cost leadership and differentiation. Cost leadership requires offering a product or service at the lowest price in its sector, while differentiation leadership occurs when a company seeks to be unique in its product mix, either through the product it offers, distribution, sales-related aspects, type of service and image, among other intangible aspects.

In this last model is where intangible assets play a key role in the generation of value, among which reputation is the most strategic asset. For this reason, both the academic and business worlds are making efforts to understand and measure reputation (9).

Although experts agree that reputation is an intangible value of great relevance for organizations, there is no consensus on the definition of the concept, nor on the factors that compose it, methods to measure it and thus determine to what extent it affects the different business indicators (10,11). Although there are advances in the creation and application of instruments that measure corporate reputation, experts have expressed the importance of creating consensus regarding key definitions in this field (12) and also highlight the need to apply new instruments that are capable of covering aspects that the current ones do not consider (13).

The aim of this study is to analyze the psychometric properties of an instrument used to measure reputation in the banking field, in its Spanish version, adapted to the healthcare field, thus determining whether it constitutes a reliable and valid instrument for use in future research on samples of users in the healthcare sector.

## Material and Methods

-Validation of the instrument Description

The instrument in question was a questionnaire, previously applied in Malaysia, for the banking field, which consists of three (14) dimensions, the first one measuring Relational Commitment, the second one measuring Behavioral Intention, and the last one measuring Relational Value, with a total of 35 questions, measured by means of a scale from 1 to 5, 5 being related to the answer “strongly agree” and 1 to “strongly disagree”, all presenting the same overall weight of the survey.

-Type of study and selected population

The study was a qualitative instrument validation study. The population consisted of patients who attended the Dental Clinic of the Andrés Bello University of Concepción (Chile), with a sample of 323 men and women, considered as the number that allowed reaching the saturation of information to apply the instrument.

-Sample inclusion criteria

An active patient of the Dental Clinic of the Universidad Andrés Bello, Concepción campus, over 18 years of age and having signed the informed consent to participate in the research. At the same time, to have understood the instructions for filling out the questionnaire, and finally to be mentally able to self-complete the survey.

-Adaptation of the reference instrument to the health care setting

The questionnaire was translated and adjusted to the healthcare setting by a panel of five professionals, three linked to the health field, one professional in the humanities and one bilingual expert.

The process of cross-cultural adjustment of the questionnaire to Spanish, created by Khan, Kadir and Wahab (15) was carried out using the methodology recommended by Ramada-Rodilla *et al*. (16) for the cultural adaptation and validation of health questionnaires, which consisted of 6 stages: language adaptation (17), synthesis of translations (11), back translation (14), adaptation to the health care setting (18), pilot study (19) and implementation of the instrument (20).

-Conducting the pilot study

A sample of 97 patients undergoing treatment at the University Health Center of the Peruvian University of Applied Sciences (Lima, Peru), attended by dental students, under the supervision of a professor, was subjected to a pilot test by applying the written questionnaire in order to evaluate the applicability and feasibility of the instrument and the quality of the translation.

Likewise, a pre-test was applied with the intention of calculating whether the instrument was sTable, this was after 20 days of the first data collection.

-Applicability and validation of the instrument obtained.

To evaluate the applicability and validation of the instrument, it was applied to a sample of 325 patients over 18 years of age who attended the Dental Clinic of the Universidad Andrés Bello de la Concepción Chile, during August and September 2019 and later between November and December 2020, whose treatments were carried out by students of the Dental School, under teaching supervision.

After 20 days, the same group of patients was contacted to remind them to answer the survey again, in order to measure the stability of the instrument. Of the patients initially studied, only 323 surveys were considered valid for the study.

-Statistical analysis.

Regarding the analytical segment, statistics were applied using SPSS 25 and Microsoft Excel 2010 statistical software.

-Pilot study phase

Two exploratory factor analyses were performed on the post-test pilot sample, one including all the questions of the questionnaire and a second excluding these questions. The analysis was performed using Bartlett’s test and the Kaiser-Meyer-Olkin coefficient (KMO).

To analyze the multifactorial variance, Principal Component Analysis (PCA) was applied as an extraction method and Varimax with Kaiser normalization was applied as a rotation method, in order to verify whether the factor analysis was adequate.

-Instrument applicability analysis

An exploratory factor analysis was applied to the final sample with the aforementioned methods.

Two confirmatory factor analyses were performed for the three dimensions studied: Relational Commitment (RC), Behavioral Intention (BI) and Relational Value (RV); the first one includes all the items and in the second one some items are excluded.

Three (3) fit indicators were applied:

SRMR Standardized root mean square residual. CFI Comparative Fit Index.

TLI Tuker-Lewis index.

-Hypotheses

The hypotheses that guided this study are as follows:

H1: The instrument proposed by Khan, Kadir and Wahab to measure reputation through the measurement of relational value in the banking sector is applicable to measure reputation in healthcare.

H2: The perception of relational value by health care users is directly related to their relational commitment.

H3: The perception of relational value by health sector users is directly related to their behavioral intention.

## Results

-Adaptation of the reference instrument to the health care setting

All the questions were reviewed by the panel of experts from different disciplines, which were individually adapted to the health care setting. In a linked session, all the already adapted questions were reviewed by means of rounds of opinion, with the mission of constructing an instrument as comprehensible as possible to be understood by children under 12 years of age. The decision to make any changes or not to the questions was taken by consensus, for which a minimum consensus of 80% or more was considered.

The pilot test was carried out in Peru, outside the study phase (Chile), taking into account that these countries share a common linguistic base and that the questionnaire was easy to understand, without idiomatic lexicons that could generate difficulties of understanding between people in different geographical locations within Spanish-speaking nations. It was observed that the patients studied showed no problems in understanding the questions, so it was not necessary to revise and readapt the instrument.

-Pilot study

All the answers of the 97 patients were considered valid, reporting 43.29% of men and 56.71% of women, with an age range between 19 and 68 years.

The results according to the analysis of the instrument proposed by Khan, Kadir and Wahab (15) to calculate reputation through the calculation of relational value in the banking sector, and its applicability to calculate reputation in the health care setting, are shown in Table 1, according to the initial factor analysis which presented all the questions of the instrument Bartlett’s test of sphericity showed that the factor analysis is the appropriate one to run (Approx. Chi-Square =5631.36, p. <.001). Likewise, the KMO coefficient reported a value of .817, making the exploratory factor analysis admissible.

**Table 1:**
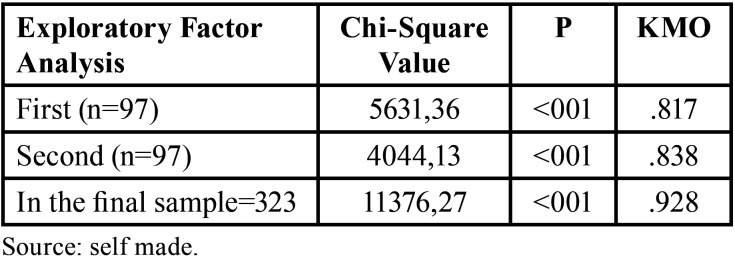
Summary Results of exploratory factor analysis (Chi-Square, p, KMO).

The results obtained through the analysis of variance in the first factor analysis showed a total explained variance of 83.984% with the use of the total number of items of the reference questionnaire (Table 2). Therefore, the factorial analysis model was composed of 6 components that explain 83.984% of the variability of the 35 items, a value that, being greater than 50.0%, is considered a tolerable percentage (21).

**Table 2:**
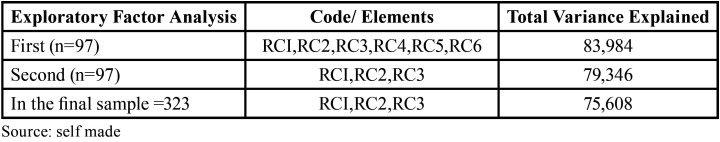
Total variance explained for the exploratory factor analyzes on the pilot sample (n=97) and the final sample (n=323).

The Principal Component Analysis for the same sample, and the results obtained with the Varimax rotation method with Kaiser normalization are shown in Table 3,  Table 3 cont., where 6 components were formed with their respective factor loadings from the 35 items. Due to this, the dimensions formulated in the reference instrument are not established with the pertinent items. Based on this, certain items were eliminated until the desired instrument was constructed from a statistical point of view.

**Table 3:**
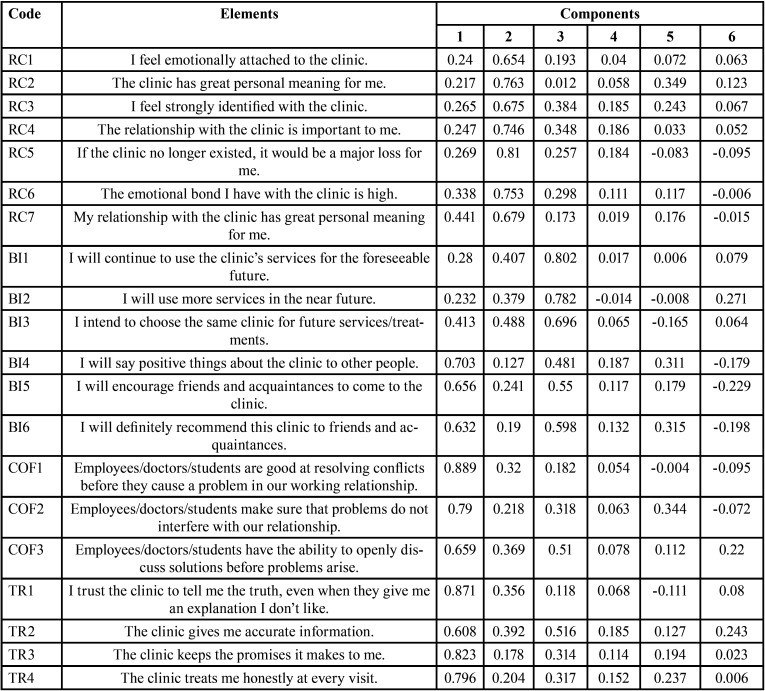
Rotated component matrix of the first exploratory factorial analysis on the pilot sample (n=97).

**Table 3 cont.**
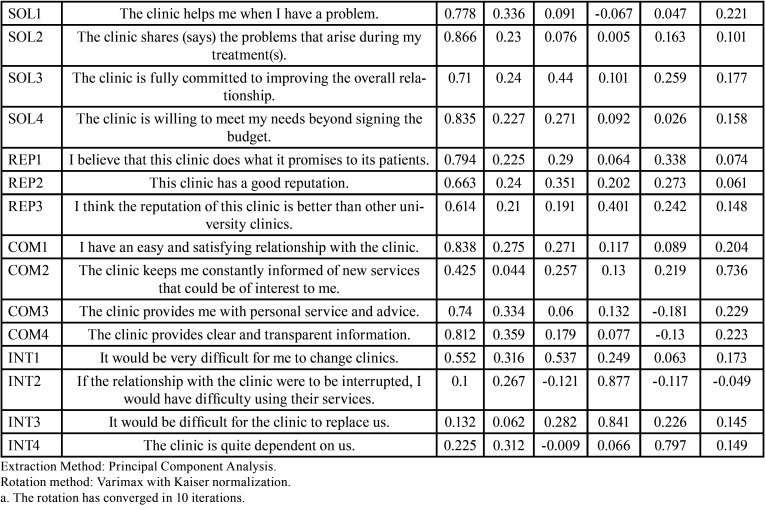
Rotated component matrix of the first exploratory factorial analysis on the pilot sample (n=97).

-Applicability of the Instrument

The results of the Factor Analysis of the final sample are shown below, with the intention of achieving a model questionnaire with the 3 dimensions that were considered in the reference instrument and that statistically was the most indicated; taking into account the results of the Principal Component Analysis previously obtained, certain items of the questionnaire were selected from the Pilot Test to perform the Second Exploratory Factor Analysis, to carry out this the following items were eliminated: BI4 I will talk to other people positively regarding the clinic, BI5 I will encourage them to come to the clinic, BI6 I will definitely recommend this clinic, COM2 The clinic keeps me constantly informed about its new services, INT1 changing clinic would be very difficult for me, INT2 I would present problems in using its services if I interrupt my relationship with the clinic, I would have problems in using its services, INT3 it is difficult for the clinic to replace us, INT4 the clinic depends on us for its operation; Once the second Bartlett’s test of sphericity was carried out, where it was shown that the second factor analysis is usable according to the data obtained through the Approx. Chi-Square = 4044.13, p. <.001. On the other hand, a KMO coefficient of .838 was obtained, so it is recommended to apply the second exploratory factor analysis.

For the second analysis, a total explained variance of 79.346% was obtained, so that the model of the second exploratory factor analysis was made up of 3 components that explain the variability of the items, being accepTable because it is greater than 50% (21).

Table 4 shows the components matrix with the Varimax rotation method without the elimination of the items mentioned above. Likewise, factor loadings are distinguished showing each item with its respective theoretical dimension. For the Relational Commitment dimension, factor loadings ranging from 0.644 to 0.804 are observed; for the Behavioral Intention dimension, factor loadings ranging from 0.742 to 0.840 are observed; and finally, for the Relational Value dimension, factor loadings ranging from 0.654 to 0.876 are observed. After the application of the instrument under the same conditions as the pilot study, the statistical analysis was applied. Thus, it is reported that, by means of the result of Bartlett’s test of sphericity, see Table 5, where the applicability of the exploratory factor analysis is deduced (Approx. Chi-Square = 11376.27, p.<.001). In turn, the KMO coefficient reinforces the idea that the application of an exploratory factor analysis is plausible.

**Table 4:**
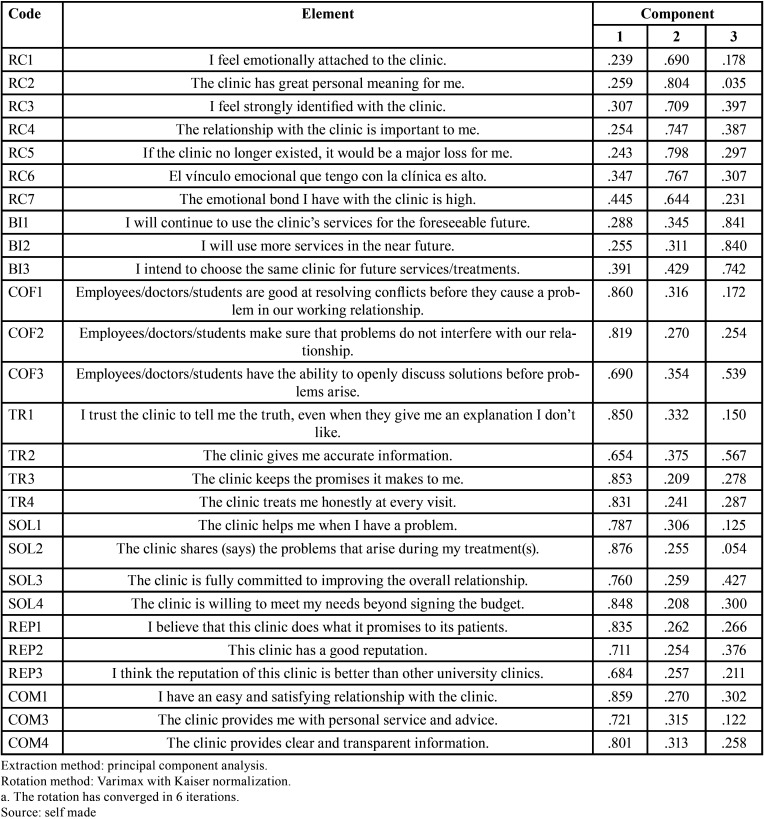
Rotated Component Matrix Of The Second Exploratory Factor Analysis Of The Pilot Sample (N=97).

**Table 5:**
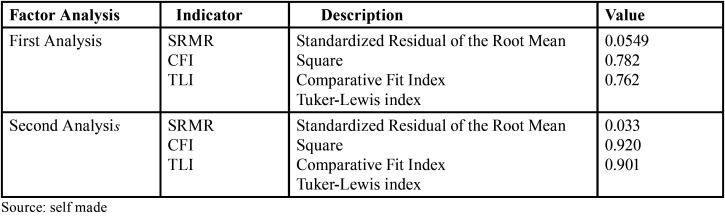
Adjustment indicators of the questionnaire for the first and second confirmatory factor analysis (n=323).

Similarly, a total variance of 75.608% was reached for the final sample; therefore, the exploratory factor analysis model presents 3 components that reveal the variability of the instrument’s items, a result that is acceptable because it is greater than 50.0% (21).

According to the results obtained through the rotated component matrix of the exploratory factor analysis, it was evidenced that the items selected by means of the factor analysis during the pilot test the respective dimensions can be adapted to each other, and that the eigenvalues for the dimension Relational Commitment (RC) range from 0. 681 to 0.807, with respect to Behavioral Intention (BI) as a dimension, eigenvalues ranging from 0.761 to 0.800 were observed and finally for the dimension Relational Value (RV) values ranging from 0.621 to 0.870 were observed.

-First confirmatory factor analysis

**Figure 1:**
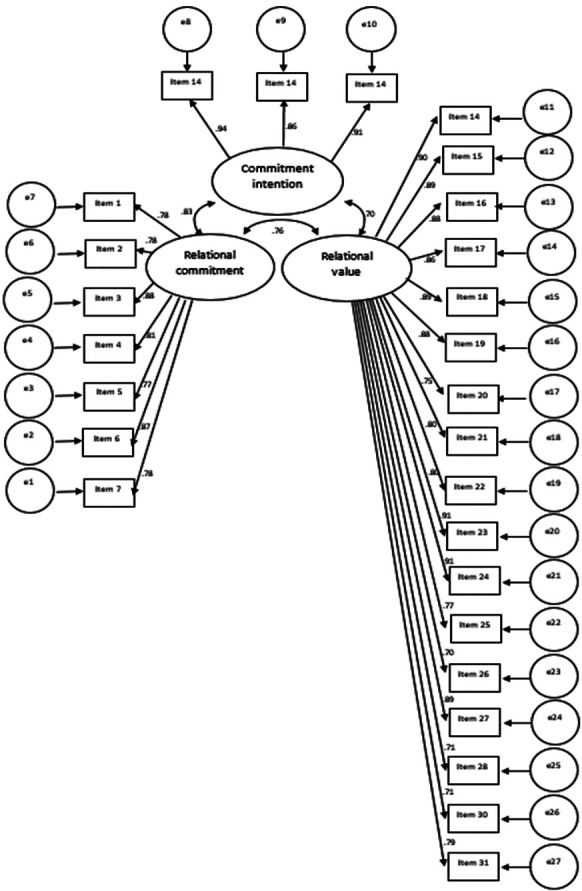
Trajectory diagram of the questionnaire for the first confirmatory factor analysis (n=323).

With respect to the first confirmatory factor analysis, it was initially possible to analyze the three (3) dimensions studied, observing in the first place, that with respect to the Relational Commitment (RC) dimension, standardized coefficients are presented in an interval of 0.78 to 0.88. Similarly, with respect to the Behavioral Intention (BI) dimension, standardized coefficients between 0.86 and 0.94 were observed. Finally, with respect to the Relational Value (RV) dimension, coefficients between 0.70 and 0.91 were reached. In all cases, coefficients with an accepTable intensity were observed, since they are values higher than 0.30, Fig. 1 (22).

**Figure 2:**
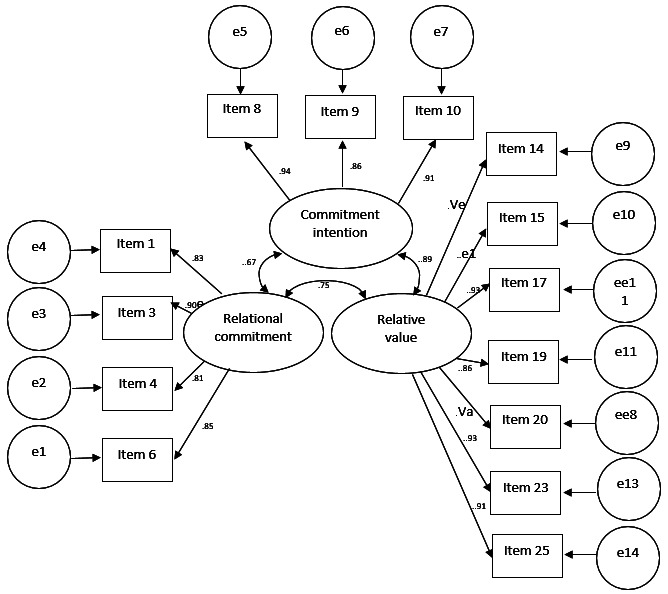
Trajectory diagram of the questionnaire for the second confirmatory factor analysis (n=323).

After the calculation of the questionnaire adjustment indicators for the first and second confirmatory factorial analysis (n=323), lower values of CFI and TLI of 0.782 and 0.762 respectively were reported in the first analysis, which are not accepTable, since they are lower than 0.90 (23). That is why a second confirmatory factor analysis was performed, filtering additional items of the questionnaire, where it was found that for this second confirmatory factor analysis there is an SRMR lower than 0.08, so that the questionnaire has a good adjustment of items, Fig. 2 (24). Likewise, regarding the comparative fit, there are CFI and TLI values of 0.920 and 0.901 respectively. Therefore, the items present an acceptable comparative fit, since they are higher than 0.90 (23).

-Correlational analysis for hypotheses 2 and 3

To corroborate the hypotheses that the perception of the relational value of the users of the dental health sector is directly related to their relational commitment (H2) and that the perception of their relational value is directly related to their behavioral intention (H3), we proceeded to the contrast of the above mentioned hypotheses following the Statistical Analysis flowchart, Therefore, Kolmogórov-Smirnov Normality tests were performed for both hypotheses in order to evaluate if there was a normal distribution in the dimensions of Relational Commitment, Behavioral Intention and Relational Value, observing that these dimensions of study did not have normality, since each one has a *p-value* lower than 0. 05 of significance, so we proceeded to use Spearman’s nonparametric test.

According to the results obtained through Spearman’s test in the evaluation of hypothesis 2 (H2), an Rho of 0.720 was obtained with a *p-value* of 0.000 (*p-value*<0.05), which shows that the perception of relational value expressed by users has a direct relationship with relational commitment, and can be considered as a strong relationship.

Similarly, for the evaluation of hypothesis 3 (H3) through Spearman’s test, a Rho coefficient of 0.686 with a *p-value* of 0.000 (*p-value*<0.05) was obtained, which demonstrates the existence of a direct relationship between relational value and behavioral intention, which can be considered a strong relationship.

## Discussion

A literature review has been carried out on the concept of corporate reputation, corporate image and corporate identity and how they are strongly present in the healthcare field. Furthermore, through the theoretical review of these terms, it has been possible to determine that reputation, as a socially constructed asset, has been gaining importance within organizations due to its increasing relevance as a strategic resource.

It is evident that reputation is an intangible, dynamic and multidimensional asset that gives value to organizations, and that its measurement not only allows comparison with other companies, but also provides valuable information to be incorporated into continuous improvement processes. Proof of the efforts of both the academic and business worlds is the large number of instruments and organizations that have emerged around reputation: Corporate Reputation Institute, Spanish Corporate Reputation Monitor, RepTrack monitor, among others.

From the literature review it can also be inferred that reputation does not correspond to the consequence of the implementation of a marketing campaign, but that reputation is built in the minds of its users and other stakeholders based mainly on their ethical behavior, thanks to which even emotional bonds are generated, such as relational value.

Relational value has been identified as a relevant factor in the construction of an organization’s reputation, relatively recently, and identifies those aspects that a user values and that contribute to establishing a long-term relationship with the organization. In the case of the healthcare sector, the relational value is more evident than in other sectors, since it generates situations with a high emotional charge that even compromise life.

The literature shows that institutions belonging to the health sector have shown a continuous interest in measuring the satisfaction of their users, thanks to which improvements are systematically implemented. There are also rankings for measuring the reputation of hospitals such as the Monitor de Reputación Sanitaria prepared by the Monitor Español de Reputación Corporativa (MERCO) or the U.S. News & World Report’s annual Best Hospital Rankings which chooses the fifty best hospitals in twelve specialties in terms of reputation in the United States.

The growing concern for improving the reputation of public health services, in terms of their results and their administrative and management policies, is evident in the implementation of measures aimed at guaranteeing equity, expanding coverage, minimizing costs, facilitating freedom for patients to select the professionals who provide services, among others, with the aim of improving the adaptation of the experience of patients and their families to their expectations. Thanks to the implementation in recent years of these measures to improve the quality of care focused on user satisfaction, it is possible to observe various examples of quality management policies and results in the reputation of health services (13).

With all this in mind, reputation management and quality improvement are processes that make it possible to respond to the needs expressed by the client, and it is possible to employ such a strategy to improve the valuation of the service, increase the demand for it and the level of satisfaction of current and potential users. The patient’s point of view has become the focus of attention. It is therefore necessary to set aside the technical limitations of the patient, and focus on promoting a maximum level of satisfaction and therefore the optimum level of service reputation (13).

There is a large body of literature providing evidence of the numerous methods of measuring the satisfaction of health users, as well as cross-cultural adaptations that allow their measurement (25), however, neither price nor satisfaction are the only indicators of corporate reputation (30).

The decision to apply the instrument created by Khan, Kadir and Wahab (15) lies in the fact that it is the first study reported in the literature in which relational value (indirect value) is measured in a company-customer context and with direct values such as satisfaction, which in turn relate it to behavioral intention, in accordance with what was proposed by Hartman in 1967, who stated that the affective and cognitive components play a fundamental role in the perception of value by customers.

The process of transcultural adaptation to Spanish of the questionnaire created by Khan, Kadir and Wahab (15) was carried out following the methodology recommended by Ramada, *et al*. (16) for the cultural adaptation and validation of health questionnaires and included 6 stages: language adaptation, synthesis of translations, back-translation, adaptation to the health care setting, pilot study and application of the instrument to the sample.

In the first phase of idiomatic adaptation, an attempt was made to maintain the structure of the original questionnaire, so that the resulting instrument would have semantic, conceptual and experiential equivalence with the original. To this end, the instrument was translated directly by two independent bilingual translators, whose mother tongue was Spanish. One of the translators was familiar with the objectives and concepts considered in the questionnaire and had previous experience in technical translation of texts from English into Spanish.

The other translator had no previous knowledge of the instrument and did not know the objectives of the study. This helped the translation to be more adjusted to a more colloquial Spanish, detecting possible comprehension and translation difficulties resulting from technical or uncommon terms.

The synthesis of translations was generated from the comparison of the translations made. The translators discussed the discrepancies between the two versions in order to reach a consensus translation.

The back-translation, or reverse translation, was performed by two other English translators, both of whom were native English speakers. Both professionals worked independently, without knowing the original version of the instrument, had no prior knowledge of reputation and were unaware of the objectives of the study. As in the translation process, both translations were reviewed by the translators, with the aim of finding discrepancies and reaching consensus.

The adaptation to the healthcare setting was carried out by a multidisciplinary committee, which arrived at a consensus version of the questionnaire, which also ensured that the instrument was sufficiently comprehensible to be understood by a 12-year-old schoolchild.

It was decided to carry out the pilot phase in Peru, outside of the study phase (Chile), on the understanding that both countries share a common linguistic substratum and that the questionnaire was sufficiently easy to understand, without idiomatic turns of phrase that could generate comprehension problems between people in different geographical locations within Spanish-speaking countries. The patients consulted did not report any difficulties in understanding the questions, so it was not necessary to revise and readapt the instrument.

This pilot phase included 97 valid questionnaires for the study, and allowed a first adjustment of the instrument, since some items were not sufficiently representative of the construct they were intended to measure, according to the original questionnaire.

In the instrument application phase, a sample of 323 questionnaires was taken, where it is important to mention that several adjustments had to be made to the applied instrument until an adequate adjustment was found for the CFI and TLI, since, as indicated by (27), the adoption of a single adjustment index to accept or reject a model can be risky and even incorrect.

The present study confirms that users of a health service perceive value through multiple aspects, including trust, conflict resolution capacity, solidarity and reputation. Therefore, being able to measure these aspects, in addition to increasing user knowledge to improve their satisfaction, could build a deeper and more lasting relationship that would not only ensure patient loyalty from an economic point of view, but would also lead to greater commitment to self-care and thus an improvement in their quality of life, as indicated in a study by Abu-Serriah, *et al* (28).

On the other hand, this study proves that there is a direct relationship between the perception of relational value and relational commitment in health care users, comparable to the results evidenced by Khan, Kadir and Wahab (15) in banking users.

Consistent with the research by Khan, Kadir and Wahab (15) and a more recent one by Lee, *et al*. (29) who studied behavioral intention in millennial travelers, the present study evidences that there is a direct relationship between relational value and behavioral intention. This relationship is particularly relevant in the current context, where the high supply of services in the health sector makes loyalty more difficult to obtain and thus the prediction of future behavior becomes more uncertain (30).

Although the present study shows that the instrument used as a reference is applicable to the dental field, it has been necessary to make a downward adjustment in the number of questions to be asked. However, this reduction in the instrument shows that it is possible to measure the relational value considering a smaller number of variables, without limiting its measurement.

According to the second confirmatory factor analysis, the subdimensions “communication” and “interdependence” should be eliminated from the instrument, leaving only the dimensions “conflict”, “trust”, “solidarity” and “reputation” as subdimensions that contribute to measuring the relational value dimension. In the same second confirmatory factor analysis there is an SRMR lower than 0.08, so that the questionnaire has a good item fit as reported in the literature. Likewise, regarding the comparative fit, there are CFI and TLI values of .920 and .901 respectively. Therefore, the items finally selected present an accepTable comparative fit, since they are higher than .90 as reported by Schermelleh-Engel, *et al*. (23).
